# Evolution of Translational Machinery in Fast- and Slow-Growing Bacteria

**DOI:** 10.3390/microorganisms14020377

**Published:** 2026-02-05

**Authors:** Xuhua Xia

**Affiliations:** 1 Department of Biology, University of Ottawa, Ottawa, ON K1N 6N5, Canada; xxia@uottawa.ca; 2 Ottawa Institute of Systems Biology, University of Ottawa, Ottawa, ON K1H 8M5, Canada

**Keywords:** *Vibrio natriegens*, *Clostridium perfringens*, *Mycobacterium tuberculosis*, *Borrelia burgdorferi*, translation efficiency, *rrn* operons, tRNA, doubling time

## Abstract

Bacterial species differ dramatically in their growth rates, reflecting distinct ecological strategies and physiological constraints. Because protein synthesis is a major determinant of cellular replication, I examined how genomic investment in the translation machinery varies across bacteria with widely different doubling times. Using 20 bacterial species spanning two major bacterial kingdoms (Bacillati and Pseudomonadati), I quantified ribosomal RNA (*rrn*) operon number, total tRNA gene number, and the allocation of tRNA genes among amino acids. Rapidly replicating *Vibrio natriegens* has 11 *rrn* operons and 129 tRNA genes in its genome, whereas slowly replicating *Borrelia burgdorferi* has only one *rrn* operon and 32 tRNA genes. I show that both the *rrn* operon number and the tRNA gene number decline sharply with increasing generation time, a pattern observed independently within each bacterial kingdom. Moreover, tRNA gene allocation is highly non-uniform: amino acids that are frequently used in proteins and encoded by large synonymous codon families are supported by disproportionately more tRNA genes. This relationship is well described by a simple model incorporating amino acid usage and codon family size, as illustrated in rapidly growing species such as *Vibrio natriegens* and *Clostridium perfringens*. In contrast, slow-growing bacteria maintain relatively minimalist translation systems. Together, these results demonstrate that bacterial genomes are systematically optimized for translation in a manner tightly coupled to growth strategy, revealing how natural selection tunes both the capacity and structure of the translation machinery.

## 1. Introduction

Bacterial species differ enormously in their capacity for growth and replication. Under favorable laboratory conditions, some bacteria, such as *Vibrio natriegens*, can divide in less than ten minutes [1,2], whereas others, including *Mycobacterium tuberculosis*, require 20–30 h to complete a single doubling [3,4,5]. These differences are not simply quantitative curiosities but reflect fundamentally distinct ecological strategies constrained by their respective environment. Rapidly growing species are often adapted to nutrient-rich, fluctuating environments where competitive advantage is gained by swift exploitation of resources, whereas slow-growing species may inhabit nutrient-poor or stable environments where persistence and stress resistance, including drug resistance and evasion of host immune defense, are more important than speed of replication. Because growth rate directly constrains how quickly genetic information can be converted into biomass, it provides a natural axis along which cellular machinery, especially the translation machinery, may be optimized.

A central determinant of growth rate in microbial species is their capacity for protein synthesis, which is true not only in bacteria [4,6,7,8,9] but also in phages [10,11,12]. Classic physiological studies have shown that fast-growing bacteria devote a large fraction of their total cellular mass to ribosomes, whereas slow-growing bacteria maintain far fewer ribosomes per cell. Ribosomes represent parallel protein-production factories, and their abundance in *Escherichia coli* increases linearly with the growth rate [13,14]. Proteins account for more than half of the cell dry mass in several enterobacterial species [15,16]. In *E. coli*, the ratio of dry weight to wet weight is 0.2294 [17], and the ratio of protein weight to wet weight is about 0.2 [18]. Thus, proteins contribute about 87% (=0.2/0.2294) of the cellular dry biomass. For this reason, the growth rate of *E. coli* increases with the rate of protein production [16]. This relationship reflects a basic biophysical constraint: protein synthesis is rate-limiting for growth when nutrients are abundant, and increasing ribosome number increases translational throughput. Conversely, when growth is limited by factors such as nutrient uptake, metabolic flux, or host immune pressure, investing heavily in ribosomes may yield diminishing returns.

Ribosomes are the molecular machines responsible for translating mRNA into protein, and their abundance in the cell is nearly perfectly correlated with bacterial doubling time [19]. If ribosome abundance is a major determinant of growth rate, then rapidly replicating bacterial species should show genomic signatures of increased investment in translational machinery. One obvious manifestation of this investment is the number of ribosomal RNA (*rrn*) operons encoded in the genome. Multiple *rrn* operons allow faster ribosome biogenesis and enable cells to rapidly scale up protein synthesis during exponential growth. In parallel, efficient translation also requires a sufficient supply of charged tRNAs to match ribosomal demand. Thus, one would expect fast-growing species not only to harbor more *rrn* genes but also to encode a larger and more redundant set of tRNA genes. In contrast, slow-growing species, whose replication may be constrained by factors other than translation, may not benefit from such redundancy and are expected to maintain a more minimal translational apparatus.

Fast-replicating bacteria have more rRNA and tRNA genes than slow-replicating ones [20], consistent with the conceptual framework developed above, although the study suffers from the shortcoming of a limited sample of bacterial species, i.e., the rapid-replicating species are Gram-negative, whereas the slow-replicating ones are Gram-positive. The two also differ much in genomic GC content, with the latter being much more GC-rich than the former.

How many tRNA genes should be encoded in a genome? At the extreme end of translational simplification are systems such as vertebrate mitochondria, which typically possess a minimal set of tRNA genes, often one per synonymous codon family, relying on extensive wobble pairing to decode all codons [21]. A similar simplification may occur in slow-replicating bacterial species such as *Mycobacterium tuberculosis* or *M. leprae*, where translational demand may be modest. Take *M. tuberculosis*, for example. Its cells build their private niches inside the host macrophage, essentially isolated from the surrounding environment. It wraps itself with a thick multilamellar architecture, including a thick layer of mycolic acids that serves two functions [22]: (1) to prevent hydrophilic antibiotics from reaching the cell membrane [23], and (2) to evade the attack of the host immune system [24]. However, after being phagocytosed by pulmonary macrophages and confined within the phagosome and the granuloma, *M. tuberculosis* survives in an extremely nutrient-limited environment and adopts a prolonged stage of dormancy with no chance of rapid growth [5]. Ribosomes are energetically expensive machines. The theory of energy optimization [25] states that it would be energetically wasteful to invest too much in cellular translation machines if the cell does not have enough energy and nutrients to operate these machines. In such cases, natural selection may favor genomic investment in survival and stress resistance in *M. tuberculosis* instead [26,27,28,29,30,31]. The same is applicable to other slow-replicating bacterial species, such as *Helicobacter pylori*, which needs to invest much in its acid-resistance cellular machinery in order to survive the acidic environment in the mammalian stomach [32,33,34,35,36,37,38,39].

In contrast, rapidly replicating bacteria often possess more complex translation machines, with multiple distinct tRNAs assigned to the same amino acid or codon family. In such systems, the number of tRNA genes associated with a given amino acid is unlikely to be arbitrary. It has long been recognized that some amino acids are decoded by more tRNAs than others, and this varies with species. For example, there are more tRNA^Ala^ genes in *Aedes aegypti* than in *Anopheles gambiae* [40].

The number of tRNA genes per amino acid should depend on at least two factors. First, amino acid usage in coding sequences should play a major role: amino acids that appear frequently in proteins impose a higher demand on the translation system and should therefore be supported by a larger pool of corresponding tRNAs [20]. Second, the size of the codon family, defined as the number of synonymous codons encoding a given amino acid, should also influence tRNA gene number. Amino acids with six-member codon families, such as leucine, arginine, and serine in the standard genetic code, necessarily need more tRNA genes than those encoded by a single codon (methionine, tryptophan) or two codons (e.g., lysine, aspartate) [41], everything else being equal.

Taken together, these considerations lead to several testable predictions. First, bacterial species with shorter doubling times should possess more *rrn* operons and more tRNA genes than slow-growing species. Second, within a genome, the number of tRNA genes should vary substantially among amino acids rather than being evenly distributed. That is, the number of tRNA genes assigned to an amino acid should increase with amino acid usage in proteins. These predictions form the basis of the analyses presented below, based on 20 bacterial species representing dramatically different doubling times from diverse bacterial lineages. Empirical evidence supports the predictions.

## 2. Materials and Methods

The 20 bacterial species included belong to two bacterial kingdoms, Bacillati (first 10 species in Table 1), which are almost all Gram-positive with *Bacillus subtilis* as the model species, and Pseudomonadati (the last 10 species in Table 1), which are almost all Gram-negative with *Escherichia coli* as a model species. Generation time has typically been quantified only in model organisms (e.g., *E. coli* as a model species for Gram-negative bacteria and *Bacillus subtilis* as a model species for Gram-positive bacteria) and very harmful pathogens, so the sample of 20 species is obviously not a random sample but was chosen to represent dramatically different generation times in the two bacterial kingdoms. Also, the optimal growth condition from an experimenter’s perspective may not be the true optimal, so the generation time in Table 1 may be subject to estimation bias. However, the rank of the relative length of generation time (Table 1) should be robust, and the subsequent analysis will mainly be based on this rank.

The RefSeq genomic sequences for the 20 species were downloaded from GenBank by using the accession numbers in Table 1. The two *Vibrio* species have two chromosomes each (Table 1). The software DAMBE 7.3.0 [66] was used to extract coding sequences (CDSs), rRNA, and tRNA genes from the GenBank files (.gbff, GenBank Flat Files). DAMBE can extract coding sequences (CDSs), upstream and downstream of coding sequences, rRNA and tRNA sequences, sequences between CDSs, and sequences upstream and downstream of CDSs. To download a long list of GenBank files of bacterial genomes, one can enter the accessions into a text file, one accession per line, and use NCBI’s Batch Entrez function to download all of them. These downloaded files are in GenBank flat file (.gbff) format, equivalent to the previous .gbk or .gb file format. They are all plain text files but contain gene annotations in the FEATURES table.

To extract all rRNA and tRNA genes in a species, say E_coli.gbff, one would open E_coli.gbff into DAMBE. In the next dialog, choose “rRNA” to extract all rRNA sequences. To download all tRNA genes, replace “rRNA” with “tRNA” in the dialog after opening the .gbff file into DAMBE. To extract all coding sequences, choose “CDS” in the dialog, and the “gene” option to use gene names as sequence IDs. In the next dialog asking for sequence type, choose protein-coding sequence and enter genetic code (translation table) 11 for bacterial species. To work on amino acid sequences, click “Sequences|Work on amino acid sequences”. DAMBE will translate the CDSs to amino acid sequences. This translation would be wrong if you specify the wrong genetic code. DAMBE implements all 27 known genetic codes.

To obtain codon frequencies in DAMBE, click “Seq.Analysis|Codon usage|Relative synonymous codon usage”. Amino acid usage was calculated from the full set of protein-coding genes for each species, ensuring that usage estimates reflect genome-wide translational demand rather than a subset of genes. This is obtained in DAMBE by clicking “Seq.Analysis|amino acid frequencies”. All subsequent analyses were based on these extracted datasets, allowing direct comparison of genomic investment in translational components across species.

## 3. Results

### 3.1. The Number of rrn Operons and tRNA Genes Decreases with Doubling Time

**Figure 1:**
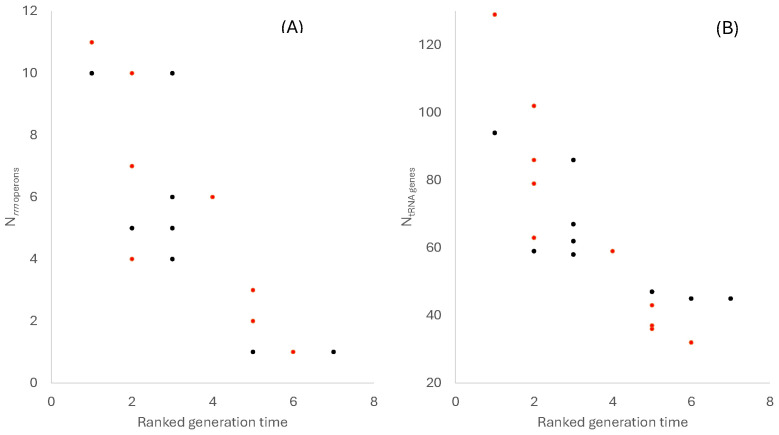
The number of *rrn* operons (**A**) and tRNA genes (**B**) decreases with the increasing generation time (ranked in Table 1). Red dots: Pseudomonadati. Black dots: Bacillati. Some points overlap.

We expect rapidly replicating species to invest more in the translation machinery. Two main components of the translation machinery are ribosomal RNA (rRNA) and transfer RNA (tRNA). rRNA genes reside in *rrn* operons, each containing the 16S, 23S, and 5S rRNAs, occasionally with tRNA genes in between, which are then processed into individual functional rRNAs [67]. *E. coli* has seven rRNA operons (*rrnA* to *rrnE*, *rrnG*, *rrnH*) with promoters that are almost identical to the −10 and −35 consensus [68,69], suggesting a high demand for rRNA molecules met by both efficient and parallel transcription of multiple rRNA operons. The production of ribosomes in *E. coli* is limited by rRNA production [70], which explains why *E. coli* maintains multiple *rrn* operons in its genome for parallel transcription. A generalization of this would lead to the prediction that rapidly replicating species should have more *rrn* operons than lowly replicating species. Across the twenty bacterial species examined, the number of *rrn* operons exhibits a strong negative association with doubling time (Figure 1A), which is taken as synonymous with generation time here and was ranked in Table 1. Fast-growing species in both Pseudomonadati and Bacillati typically possessed multiple *rrn* operons, whereas slow-growing species encoded far fewer, with the minimum constrained to be one (Figure 1A). This constraint should be apparent because an organism needs at least one *rrn* operon for translation. It is rRNAs that form the core of ribosomes, with all important sites such as A, P, and E sites, and ribosomal proteins padding the surface of a ribosome [71], so at least one *rrn* operon is needed.

I wish to highlight the point that the negative association between the number of *rrn* operons and the generation time was observed independently within each bacterial kingdom (Figure 1). This suggests that the relationship between *rrn* operon number and generation time reflects a general physiological principle rather than a lineage-specific peculiarity, substantiating previous proposals on the relationship between rRNA abundance and growth rate [72].

More ribosomes require more tRNA molecules [73]. A similar pattern was observed for tRNA genes (Figure 1B). Species with short doubling times encoded substantially more tRNA genes than slow-growing species, again in both bacterial kingdoms. The rapid-replicating *Vibrio natriegens* in Pseudomonadati and *Clostridium perfringens* in Bacillati feature 129 and 94 tRNA genes, respectively, whereas the slow-replicating *Borrelia burgdorferi* in Pseudomonadati and *Mycobacterium leprae* in Bacillati have only 32 and 45 tRNA genes, respectively. This finding supports the notion that translational efficiency in fast-growing bacteria depends not only on ribosome abundance but also on maintaining a sufficiently large and diverse tRNA pool.

**Figure 2:**
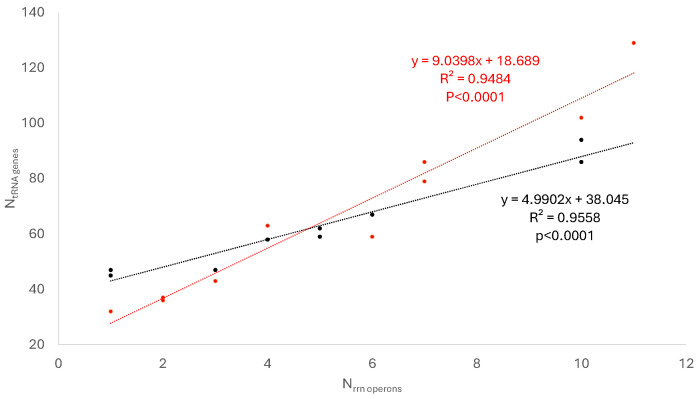
The number of tRNA genes increases with the number of *rrn* operons. Red dots: Pseudomonadati. Black dots: Bacillati.

The number of tRNA genes increases with the number of *rrn* operons in both Pseudomonadati and Bacillati (Figure 2), which could be inferred roughly from the pattern in Figure 1. However, the tight relationship between the two in both bacterial kingdoms is surprising, almost suggestive of a stoichiometric relationship.

While the plots in Figure 1 facilitate the visualization of the negative association between generation time and the two key components of the translation machinery (rRNA and tRNA), the biological meaning of the ranked generation time is not clear. As an alternative, I have used the middle value of the generation time in Table 1 as an approximate estimate of the generation time. This allows me to fit the following two equations with real biological meanings:

(1) $$N_{rrn}=1+\alpha e^{-\beta T}$$

(2) $$N_{tRNA}=\gamma +\alpha e^{-\beta T}$$

where *N_rrn_* is the number of *rrn* operons, *N_tRNA_* is the number of tRNA genes, and *T* is the generation time in minutes. Equation (1) ensures a minimum *N_rrn_* of 1 because no bacterial species can survive without at least one *rrn* operon. Equation (2) ensures a minimum *N_tRNA_* of γ, which could be as low as 22, as observed in vertebrate mitochondria.

The parameters of the models, estimated by the likelihood method separately for Bacillati and Pseudomonadati, are shown in Table 2. To perform a likelihood ratio test (LRT), one calculates the likelihood ratio chi-square, often represented by G^2^, with G^2^ = 2 × (lnL_Model_ − lnL_null_). In the LRT for *rrn*, the model is in Equation (1) and the null model is N_rrn_ = C (where C is the mean of Nrrn, i.e., N_rrn_ does not depend on T). In the LRT for N_tRNA_, the model is specified in Equation (2), and the null model is N_tRNA_ = C (where C is the mean of N_tRNA_, i.e., N_tRNA_ does not depend on T).

The relationship between *N_rrn_* and *T* is similar for Bacillati and Pseudomonadati, as one would expect from Figure 1A, with *N_rrn_* spanning a similar range of values. In contrast, the relationship between *N_tRNA_* and *T* differs substantially between Bacillati and Pseudomonadati (Figure 1B), where *N_tRNA_* ranges from 45 to 94 in Bacillati but from 32 to 129 in Pseudomonadati. This is partially visible in the relationship between *N_tRNA_* and *N_rrn_* (Figure 2).

### 3.2. Association Between tRNA Genes and Amino Acid Usage

While it seems straightforward to understand why the replicating *Vibrio natriegens* should have more tRNA genes than *Borrelia burgdorferi*, it is less clear which amino acid should have more tRNA genes than others within each genome. Take *Vibrio natriegens*, for example, should its 129 tRNA genes be distributed evenly among the 20 amino acids, or should some amino acids have more tRNA genes than others?

There are three factors that could affect the distribution of tRNA genes among the 20 amino acids. First, amino acids Leu, Arg, and Ser have six synonymous codons in the standard genetic code, in contrast to Trp, which is encoded by a single codon. One needs at least two different tRNAs to decode a six-codon family, but only one tRNA for a single-codon tRNA family. Thus, the number of tRNA genes per amino acid within a species, *M_tRNA_* (to distinguish from *N_tRNA_,* which is the number of tRNA genes per genome) should increase with the synonymous codon family size (CFS, i.e., CFS = 1 for Trp and Met, but CFS = 6 for Leu, Ser, and Arg in the standard genetic code). In short, *M_tRNA_* ∝ CFS.

The second contributor to *M_tRNA_* is the functional requirement. Take amino acids Met and Trp, for example. Although both have CFS = 1 in the standard genetic code, Met is the initiation amino acid and is often the limiting factor in translation [74,75], so it has more tRNAs to carry it than Trp. The *Clostridium perfringens* genome has six tRNA^Met^ genes, but only two tRNA^Trp^ genes. Similarly, the *Vibrio natriegens* genome has 10 tRNA^Met^ genes, in contrast to only two tRNA^Trp^ genes. In fact, the *V. natriegens* genome encodes more tRNA genes for Met than for Ser, which has six synonymous codons. This implies that, if we wish to evaluate the relationship between *M_tRNA_* and CFS, we should not include Met because of its unique function as an initiation amino acid.

The third factor that would affect *M_tRNA_* is how frequently an amino acid is used in proteins. Amino acids with the same CFS, such as Glu and Cys, each with CFS = 2, can differ greatly in their usage in proteins. Glu is typically used much more frequently than Cys, so we would expect more tRNAs to transport Glu than Cys. In the coding sequences in the *Vibrio natriegens* genome, there are 95,956 Glu codons, but only 15,568 Cys codons. It seems obvious that more tRNAs (and consequently more genes, assuming equal transcription of different tRNA genes) are needed to decode the 95,956 Glu codons than the 15,568 Cys codons. Let *N_AA_* be the number of amino acids encoded by all the coding sequences in a genome. We would predict that *M_tRNA_* ∝ *N_AA_.* The simplest model incorporating the effect of both CFS and *N_AA_* on *M_tRNA_* is

(3) $$M_{tRNA}=\beta_{0}+\beta_{1}N_{AA}+\beta_{2}CFS$$

I chose the fastest replicating species in Bacillati and Pseudomonadati, i.e., *Clostridium perfringens* and *Vibrio natriegens*, respectively, to evaluate the model in Equation (3), i.e., whether *N_AA_* and CFS have a statistically significant effect on *M_tRNA_*. The results (Table 3) are consistent with the expected relationship in Equation (3). Note that we have just outlined three factors contributing to the variation in *M_tRNA_*, but Equation (3) included only two factors, i.e., *N_AA_* and CFS, because we have already singled out the initiation amino acid Met. Consequently, Table 3 includes only 19 amino acids without Met.

The regression model shows that both *N_AA_* and CFS have significant effects on *MtRNA* (Table 4). First, the global ANOVA tests showed that the model can account for a significant proportion of variation in *MtRNA* (63.6% for Pseudomonadati and 48.4% for Bacillati, Table 4). Second, the two variables, *N_AA_* and CFS, have significant and independent contributions to *MtRNA*, as revealed by the parameter-specific *t*-tests (Table 4). These results demonstrate the power of molecular biology knowledge in predicting the details of the translation machinery in diverse bacterial species.

## 4. Discussion

### 4.1. Optimizing Selection on Bacterial Translation Machinery

The results presented here reinforce the view that bacterial genomes bear clear signatures of optimization for growth rate. Genomic investment in the translation machinery, as reflected by *rrn* and tRNA gene numbers, scales predictably with doubling time across deeply divergent bacterial lineages (Table 1, Figure 1 and Figure 2). This pattern underscores the central role of translation as a limiting step in cellular replication when environmental conditions permit rapid growth.

Beyond the well-established relationship between the number of *rrn* operons and growth rate, my analyses highlight the importance of tRNA gene allocation as a second layer of translational optimization (Table 2, Table 3 and Table 4). Rather than maintaining a uniform or minimal tRNA repertoire, fast-growing bacteria selectively expand tRNA gene numbers for amino acids that are heavily used and difficult to decode efficiently due to large codon families. This suggests that selection acts not merely on total translational capacity but on the fine structure of the decoding system itself. The number of *rrn* operons primarily determines total translational capacity, whereas tRNA gene allocation shapes how efficiently that capacity is deployed across different amino acids and codon families.

In contrast, slow-growing bacteria appear to operate closer to a minimal translation system, resembling in some respects the streamlined machinery found in vertebrate mitochondria. In such organisms, growth is likely constrained by ecological or physiological factors other than translation, reducing the selective advantage of maintaining optimal composition of tRNA genes or multiple *rrn* operons. Genome economy and energetic efficiency may therefore dominate over maximal translational throughput.

Optimization involves (1) the optimization of the translation machinery and (2) the optimization of the mRNA. The classical work focused mainly on the mRNA, such as optimizing the Shine–Dalgarno sequence [76,77,78,79,80,81] or Kozak consensus [82,83] to optimize translation initiation [84,85,86], optimize codon usage to increase the efficiency of translation elongation [7,41,87,88,89,90,91,92,93,94], optimize termination signal to increase translation termination efficiency [84,94,95], and optimize the mRNA stability by modifying the 3′UTR [94]. This paper focuses on the often-neglected part of optimizing the translation machinery.

Taken together, these findings support a view of the bacterial translation machinery as an evolvable system shaped by ecological context and life-history strategy. Rather than a one-size-fits-all solution, nature has produced a spectrum of translation systems, from minimalist to highly redundant, each optimized for the demands imposed by growth rate and environmental opportunity. These findings complement classical work on codon usage bias by showing that genomic optimization extends beyond codon choice to the genomic organization of the decoding machinery itself.

### 4.2. What Is the Optimal Number of tRNAs per Ribosome?

That the tRNA availability limits translation elongation efficiency is long known [96,97,98,99,100]. Different species often have different ribosome and tRNA gene combinations. The translation in the budding yeast involves 200,000 ribosomes and 3,000,000 tRNAs [101]. Thus, there are, on average, about 15 tRNAs for one ribosome. Interestingly, this ratio is similar to the ratio of *N_tRNA_*/*N_rrn_* in rapidly replicating species. For example, the *Vibrio natriegens* genome has 11 *rrn* operons and 129 tRNA genes in its genome, so the ratio is 129/11 ≈ 12. Of course, this ratio would not be applicable for slowly replicating species with only one *rrn* operon, because a minimum of about 40 different tRNAs are needed to decode the 61 sense codons.

### 4.3. Why Do Some Bacteria Invest So Little in Translation Machinery?

One may ask why the slowly replicating bacteria should invest so little in their translation machinery. For example, *Mycobacteroides abscessus*, *Mycobacterium tuberculosis*, *M. leprae*, and *Borrelia burgdorferi* each have only one *rrn* operon and relatively few tRNA genes. Can they not invest more in their translation machinery and shorten their doubling time, thereby increasing their fitness?

Two factors would limit the genomic investment in the translation machinery in these slowly replicating species. First, the fitness of some bacteria is more related to stress resistance (e.g., antibiotic resistance and evasion of host immune defense) than to efficient translation [102]. For example, *Mycobacterium tuberculosis* needs to wrap itself around with a thick and dense multilayer architecture to protect itself against antibiotics [103], but this layered structure also restricts nutrient and oxygen exchange. The permeability coefficient for glucose is only 2.8.nm/s for a congeneric species, *M. chelonae* [104], which is five orders smaller than that for *E. coli* [105]. In this trade-off between survival and rapid replication, natural selection has apparently chosen survival over rapid replication [106].

Second, mutations still occur during this latent stage of dormancy in *M. tuberculosis* [107,108] in both protein-coding genes [109] and rRNA genes [110,111]. Thus, mutations, likely coupled with relaxed natural selection, tend to result in a suboptimal translation machinery in *M. tuberculosis.* Whether this reduced translational investment reflects relaxed selection, active selection against rapid growth, or both remains difficult to disentangle, but the genomic outcome of a minimalist translation system appears shared by slowly replicating bacterial species. Such a minimalist translation machinery is not expected to perform efficient translation even when the bacteria are not nutrient-limited. A comprehensive effort to optimize the growth condition of *M. tuberculosis* can reduce the doubling time to 14.7 h, but no further [112]. This is in dramatic contrast to *E. coli,* which experiences rapid alternation of feast and famine cycles several times each day, with natural selection eliminating those mutants that cannot translate efficiently during the feast period.

### 4.4. The Number of tRNA Genes May Not Reflect Cytoplasmic tRNA Abundance

My model in Equation (3) assumes that *N_tRNA_* and *M_tRNA_* are good proxies of tRNA abundance in the bacterial cytoplasm. While the model fits the empirical data well, there is evidence that, at least for some tRNA genes, the number of tRNA genes may not be proportional to tRNA abundance. For example, some tRNA genes are within *rrn* operons. They are likely to be transcribed more efficiently than tRNA genes outside *rrn* operons. The situation is even more complicated in eukaryotes because tRNA genes are often found within retroelements [113] and because many tRNA “genes” are silent [114]. For this reason, one should use transcriptomic data to quantify the actual cellular abundance of individual tRNA, as has been done before [115]. Alternatively, one may measure the signal strength of tRNA promoters, which are of the σ^70^-type, by using position-weight matrix [116]. If different tRNA promoters differ much in position-weight matrix score, then they tend to be differentially transcribed. Thus, tRNA gene number should be viewed as a genomic indicator of translational investment rather than a direct index of tRNA abundance, an important distinction that opens the door for integrating genomic and transcriptomic data in future studies.

Importantly, these considerations are unlikely to alter the qualitative relationships observed here, which are driven by large differences within and among species rather than fine-scale variation among individual tRNA genes. Given the taxonomic diversity in the studied species in the paper, the logic and rationale underlying the findings in this paper are likely generalizable to all bacterial species living in diverse environments.

### 4.5. Complications in Lifestyle

The 20 species in Table 1 include free-living and parasitic species, and one might ask if this difference should be taken into consideration in the comparisons. The comparison needs to be phylogeny-based. That is, given a common ancestor with two daughter lineages, one remaining free-living and the other having switched to parasitic, what are the differences in their genomic investment in the translation machinery and their replication rate? *Vibrio natriegens* is free-living, has 11 sets of *rrn* operons, and can replicate once every 10 min. Its sister lineage, *V. cholerae*, is an occasional pathogen, has 10 sets of *rrn* operons, and replicates more slowly (Table 1), but still quite fast. Within mycobacteria, the free-living *M. smegmatis* has three sets of *rrn* operons and replicates once every two hours. In contrast, the pathogenic *M. mycobacterium* and *M. leprae* each have only one set of *rrn* operon and replicate once per day (*M. tuberculosis*) to once per week (*M. leprae*).

I should emphasize that the impact of parasitism on the regulation and optimization of the three essential biosynthetic processes (genome replication, transcription, and translation) has been an underappreciated topic, partly due to the complexity involved. For example, one might expect an intracellular parasite to gain access to many nutrients in the host, including glucose, nucleotides, and amino acids. This expectation is substantiated by the genome reduction in many parasitic bacteria. *Rickettsia prowazekii* and *Chlamydia trachomatis* both have much-reduced genomes, and they have lost all enzymes for nucleotide synthesis and most enzymes for amino acid synthesis. However, *Mycobacterium tuberculosis* does not follow this rule. It wraps itself around with a thick layer of mycolic acids to form an essentially isolated “castle” with extremely limited nutrients. Even glucose has difficulty diffusing through this thick and dense layer of mycolic acid [102,104,105]. Therefore, *M. tuberculosis* would need to keep all the enzymes for nucleotide and amino acid synthesis. Another complication is the host tropism, with some bacterial pathogens specialized in a specific host, e.g., *Streptococcus pneumoniae* parasitizes the human host primarily, but others have a wide range of hosts, e.g., *Staphylococcus aureus* can occasionally parasitize not only mammalian species but also birds. The third complication is the time since the switch from a free-living to a parasitic lifestyle. For example, *Shigella flexneri* is a derived species from one of the *E. coli* lineages. It is an intracellular parasite. However, because of its very recent origin, its genome is nearly identical to that of *E. coli*. In contrast, *M. smegmatis* and *M. tuberculosis* have diverged so much that they have recently been placed in different genera. Their genome lengths are 6,993,871 bases and 4,411,532 bases, respectively. These complications would demand at least 100 species for a fair comparison to reveal the effect of parasitism on genome reduction and reorganization.

## 5. Conclusions

This study demonstrates that bacterial translation machinery is not merely scaled up or down with growth rate but is structurally optimized in a predictable and biologically interpretable manner. Fast-growing bacteria invest heavily in both ribosome biogenesis and tRNA diversity, whereas slow-growing species maintain relatively minimalist translation systems consistent with ecological constraints that favor persistence over rapid replication. Importantly, tRNA gene allocation reflects translational demand at the level of individual amino acids and codon families, revealing a fine-grained optimization that parallels, but is distinct from, codon usage bias. These findings highlight translation as a central axis of bacterial life-history evolution and underscore how genomic architecture encodes long-term adaptive strategies shaped by ecological opportunity and constraint.

## Figures and Tables

**Table 1:** Twenty species with complete genomes and well-documented generation times (GT) under optimal growth conditions. The first 10 belong to the Bacterial Kingdom Bacillati, and the last 10 belong to the Pseudomonadati.

Species	Accession ^(1)^	OGT ^(2)^	GT ^(3)^	Rank ^(4)^	Ref. ^(5)^
*Clostridium perfringens*	NZ_CP065681	43 °C	~7–15 min	1	[42,43]
*Staphylococcus aureus*	NC_007795	37 °C	~21–35 min	2	[44]
*Bacillus subtilis*	NC_000964.3	37 °C	~30–70 min	3	[45]
*Streptococcus pneumoniae*	NZ_LN831051	37 °C	~30–60 min	3	[46]
*Listeria monocytogenes*	NC_003210	37 °C	~45–60 min	3	[47,48]
*Lactobacillus plantarum*	NZ_CP028221	37 °C	~50–70 min	3	[49,50]
*Mycolicibacterium smegmatis*	NZ_CP054795.1	37 °C	~2 h	5	[51]
*Mycobacterioides abscessus*	NZ_CP034181.1	36 °C	~4–5 h	5	[52]
*Mycobacterium tuberculosis*	NC_000962.3	37 °C	~20–30 h	6	[3,4,5]
*M. leprae*	NZ_CP029543.1	30 °C	~7 days	7	[4,53]
*Vibrio natriegens*	NZ_CP009977, NZ_CP009978.1	37 °C	~10 min	1	[1,2]
*Vibrio cholerae*	NZ_CP043554, NZ_CP043556.1	37 °C	~16–20 min	2	[54]
*Escherichia coli*	NC_000913.3	37 °C	~20–30 min	2	[55]
*Pseudomonas aeruginosa*	NC_002516	37 °C	~25–30 min	2	[56,57]
*Salmonella enterica*	NC_003197	37 °C	~20–30 min	2	[57,58]
*Haemophilus influenzae*	NZ_CP007470.1	37 °C	~103–107 min	4	[59]
*Chlamydia trachomatis*	NC_000117	35–37 °C	~1.8–4.6 h	5	[60,61]
*Helicobacter pylori*	NZ_AP026446	37 °C	~2.5–3 h	5	[62]
*Campylobacter jejuni*	NC_002163	42 °C	~2–3 h	5	[63]
*Borrelia burgdorferi*	NZ_ABCW02000001 ^(6)^	33 °C	~8.3–24 h	6	[64,65]

^(1)^ GenBank accession number. ^(2)^ Optimal growth temperature. ^(3)^ Generation time under optimal growth conditions. ^(4)^ Ranking of generation time from smallest to largest. ^(5)^ References pertaining to the generation time. ^(6)^ one of the four contigs, the other three being NZ_ABCW02000002 to NZ_ABCW02000004.

**Table 2:** Estimated parameters for the models in Equations (1) and (2) by the maximum likelihood methods, shown separately for Bacillati and Pseudomonadati.

Model	α	β	γ	lnL_model_ ^(1)^	lnL_null_ ^(2)^	*p* ^(3)^
N_rrn.Bacillati_	9.000380	0.011936		−19.5164	−25.8209	0.000384
N_rrn.Pseudomonadati_	8.863043	0.010691		−18.9248	−26.0992	0.000152
N_tRNA.Bacillati_	51.377406	0.018713	45.392728	−36.4670	−151.0943	0.000000
N_tRNA.Pseudomonadati_	157.323365	0.056354	40.954750	−131.4144	−475.6256	0.000000

^(1)^ Log-likelihood for the models in Equations (1) and (2). ^(2)^ log-likelihood for the null model of no relationship between N_rrn_ or N_tRNA_ and T (generation time). ^(3)^ *p*-value from likelihood ratio tests.

**Table 3:** The counts of the 20 amino acids in all coding sequences, together with the number of codon family size (CFS) and the number of tRNA genes per amino acid (*M_tRNA_*) in *Vibrio natriegens* and *Clostridium perfringens*.

	*Vibrio natriegens*			*Clostridium perfringens*		
AA ^(1)^	N	CFS	M_tRNA_	N	CFS	M_tRNA_
A	126,982	4	7	49,128	4	4
C	15,568	2	4	10,316	2	2
D	80,754	2	6	50,881	2	3
E	95,956	2	6	74,540	2	4
F	60,938	2	4	42,363	2	4
G	103,326	4	11	60,495	4	12
H	32,846	2	2	11,807	2	2
I	92,838	3	5	87,449	3	4
K	76,752	2	4	85,533	2	7
L	151,573	6	17	85,534	6	9
N	61,206	2	5	59,147	2	4
P	58,807	4	3	24,866	4	3
Q	64,988	2	6	18,015	2	3
R	65,549	6	11	30,389	6	7
S	97,945	6	7	56,898	6	5
T	79,535	4	7	42,052	4	5
V	107,453	4	6	59,549	4	4
W	18,772	1	2	6536	1	2
Y	44,625	2	6	36,940	2	3

^(1)^ Methionine is excluded because of its special function as the initiation amino acid in protein synthesis.

**Table 4:** ANOVA tests of the model significance and individual *t*-test of parameter significance, based on Equation (3), for Pseudomonadati (A) and Bacillati (B).

Taxon		DF	SS	MS	F	*p*
(A)	Model	2	144.85534	72.42767	13.99081	0.00031
	Residual	16	82.82887	5.17680		
	Total	18	227.68421			
(B)	Model	2	57.41445	28.70722	7.50306	0.00503
	Residual	16	61.21713	3.82607		
	Total	18	118.63158			
		**β_i_**	**SE**	**T**	***p***	
(A)	Intercept	−0.27790	1.34781	−0.20619	0.83925	
	N_AA_	0.00004	0.00002	2.17467	0.04500	
	CFS	1.04273	0.43905	2.37500	0.03039	
(B)	Intercept	0.31282	1.19011	0.26285	0.79602	
	N_AA_	0.00004	0.00002	2.23869	0.03974	
	CFS	0.72833	0.30624	2.37830	0.03019	

## Data Availability

The original contributions presented in this study are included in the article. Further inquiries can be directed to the corresponding author.
